# Enhanced Dispersion of TiO_2_ Nanoparticles in a TiO_2_/PEDOT:PSS Hybrid Nanocomposite via Plasma-Liquid Interactions

**DOI:** 10.1038/srep15765

**Published:** 2015-10-26

**Authors:** Yazi Liu, Dan Sun, Sadegh Askari, Jenish Patel, Manuel Macias-Montero, Somak Mitra, Richao Zhang, Wen-Feng Lin, Davide Mariotti, Paul Maguire

**Affiliations:** 1 School of Chemistry and Life Science, Nanjing University Jinling College, Nanjing 210089, PR China; 2 Department of Chemical Engineering, Curtin University, G.P.O. Box U1987, Perth, Western Australia 6845, Australia; 3 School of Mechanical and Aerospace Engineering, Queen’s University Belfast, UK BT9 5AH; 4 Nanotechnology and Integrated Bioengineering Centre (NIBEC), University of Ulster, BT37 0QB, UK; 5 Department of Chemical Engineering, Case Western Reserve University, Cleveland, Ohio, 44106-7217, USA; 6 Department of Chemical and Biological Engineering, Zhejiang University, Hangzhou, 310002, PR China; 7 Department of Chemical Engineering, Loughborough University, Loughborough, Leicestershire, LE11 3TU,UK

## Abstract

A facile method to synthesize a TiO_2_/PEDOT:PSS hybrid nanocomposite material in aqueous solution through direct current (DC) plasma processing at atmospheric pressure and room temperature has been demonstrated. The dispersion of the TiO_2_ nanoparticles is enhanced and TiO_2_/polymer hybrid nanoparticles with a distinct core shell structure have been obtained. Increased electrical conductivity was observed for the plasma treated TiO_2_/PEDOT:PSS nanocomposite. The improvement in nanocomposite properties is due to the enhanced dispersion and stability in liquid polymer of microplasma treated TiO_2_ nanoparticles. Both plasma induced surface charge and nanoparticle surface termination with specific plasma chemical species are proposed to provide an enhanced barrier to nanoparticle agglomeration and promote nanoparticle-polymer binding.

The formation of nanocomposites, through nanoparticle (NP)—liquid polymer routes, and nanofluids, through establishing stable suspensions of NPs in liquid, offers considerable opportunities to design new materials and functionalities for a wide range of applications. Taylor *et al.*^1^ provided a detailed review of nanofluids as heat/mass transport materials, phase-change materials, electromagnetically active media, catalysts, and their application in medicine for cancer treatment, imaging and fluid motion control. Polymer nanocomposites are under active investigation for ultra-high energy capacitors^2^, coupled organic–inorganic nanostructures^3^ and stimuli-responsive polymer materials^4^. Traditional fibre-reinforced composites have been extensively studied for mechanical applications. Hybrid nanocomposites have recently attracted considerable attention in high-value-added areas such as energy storage, optical sensors, biomedical material/devices^5^,^6^. Their markedly enhanced electrical and thermal conductivity, optical and dielectric response as well as mechanical properties are mainly due to the interfacial “third phase” present between the particle and polymer matrix, enhanced by the high surface-to-volume ratio of NPs^5^.

A colloidal suspension, whether as a final step in nanofluid formation or the interim step towards a polymer nanocomposite is required to be stable, via Brownian motion, against settling. However, nanofluids are thermodynamically unstable systems and achieving kinetic stability is a necessity^7^. Frequent particle – particle contact often leads to agglomeration with agglomerate sizes up to several microns^8^ which compromises material performance. Typically, high intensity sonication is used to break up and reduce such agglomerates while chemical additives, e.g. surfactants, are introduced to avoid re-agglomeration^9^ or polymer coating is used to encourage steric hindrance^10^. The addition of stabilisation agents adds to the fluid complexity with regard to subsequent processing and can have detrimental impact on the required NP properties. It often leads to other problems such as complicated processing procedures, undesirable contamination or irreversible deterioration of the surfactant at modest temperatures^1^. Other methods of stabilisation include electrostatic repulsion where the NPs acquire surface charges once in solution^10^. However high ionic strength and strongly acidic or basic solutions are often required which can impact on processing and compromise fluid/film properties.

The most promising route to stable dispersion of nanomaterials is considered to be via either direct synthesis in liquid or ‘sequential synthesis–delivery to liquid’ approaches which limit the possibilities for native particle–particle interactions and hence avoid the need for additives and restrictive process windows. Low pressure plasma enhanced CVD coating of NPs with organic layers has been used^11^ to modify the NP properties and more recently has been shown to improve dispersion characteristics and limit agglomeration^12^. While *in situ* sequential NP synthesis followed by polymer coating has been demonstrated^13^, the use of vacuum plasmas is known to cause difficulties in material collection and handling i.e. scraping from the deposition substrate. To overcome this issue, we have demonstrated direct NP synthesis at atmospheric pressure using a non-thermal equilibrium (low temperature) plasma thus opening the possibilities of direct NP synthesis and delivery to polymer^14^. Direct synthesis of highly dispersed NPs in liquid through exposure of the liquid to a plasma has also been achieved^15^. Here the NP dispersion was maintained for many months due to plasma-induced non-equilibrium reactions in the liquid that are as yet not fully elucidated and the subject of on-going investigation^15^.

In this work, we report on the plasma treatment at atmospheric pressure and resulting characteristics of a ceramic NP–polymer composite comprised of TiO_2_ and PEDOT:PSS. The latter is the dominant transparent conductive polymer and a potential replacement for ITO in low cost polymer optoelectronic and solar cell technologies. Nanomaterial incorporation (e.g. graphene, nanotubes, SnO_2_, metal, and ceramics including TiO_2_) into PEDOT:PSS has been attempted in order to improve its optoelectronic properties. TiO_2_ is of course an important optoelectronic material^16^ with a wide variety of applications including those involving incorporation into polymer matrices for solar cells^17^, dielectrics^18^, gas sensors^19^ etc. However the dispersion and agglomeration of nano-sized TiO_2_ particles in aqueous solutions is particularly problematic^20^ and the conventional method for fabricating titania–polymer nanocomposites often results in the agglomeration or the segregation of TiO_2_ particles^21^. Since the electrical and optical properties of native PEDOT:PSS are expected to be sensitive to the effectiveness of NP incorporation, our approach offers a model system for exploring plasma – liquid – nanoparticle interactions as a route to high performance nanofluids and nanocomposites. In this work we investigate the effect of atmospheric pressure plasma treatment on TiO_2_ NP–polymer (PEDOT:PSS) colloids and the resultant NP dispersion and properties of the final nanocomposite films. We have focussed on high NP loading as applicable for example in dye sensitized solar cell (DSSC) counter electrodes or polymer LEDs where the film optical properties and/or catalytic activity are also important. We chose a demanding NP loading fraction of ~75% wt. in order to establish that our plasma technique is capable of NP dispersion at challenging and practical NP loading values.

## Methods

PEDOT–PSS (1.3 wt% dispersion in H_2_O) and TiO_2_ NPs (P25, anatase) with average primary particle size of 25 nm were purchased from Sigma-Aldrich. These NPs are widely used as a standard in the research community and in particular for photocatalysis applications^22^. The as-received TiO_2_ NPs were highly agglomerated, as is usual with such materials, and the agglomerate size ranged from 10 to 25 μm despite sonication in ethanol for 30 min (see supplementary information, S1). The films were prepared as follows: TiO_2_ NPs (10 mg) were added to 24.99 g of distilled water followed by sonication (100 W) for 30 min. PEDOT:PSS was sonicated (100 W) for 30 min and then filtered (Millipore 0.2 μm diameter), 253 μL of the filtered polymer was then added to distilled water (25 mL), the mixture was magnetically stirred for 30 min before further processing/testing. The TiO_2_/distilled water was added to this diluted polymer solution and the mixture was magnetically stirred for 1 hour to produce a TiO_2_/PEDOT:PSS/water colloid. Films were obtained by drop-casting the colloid mixture onto substrates, followed by drying in air for 6 hours at 25 °C.

**Figure 1:**
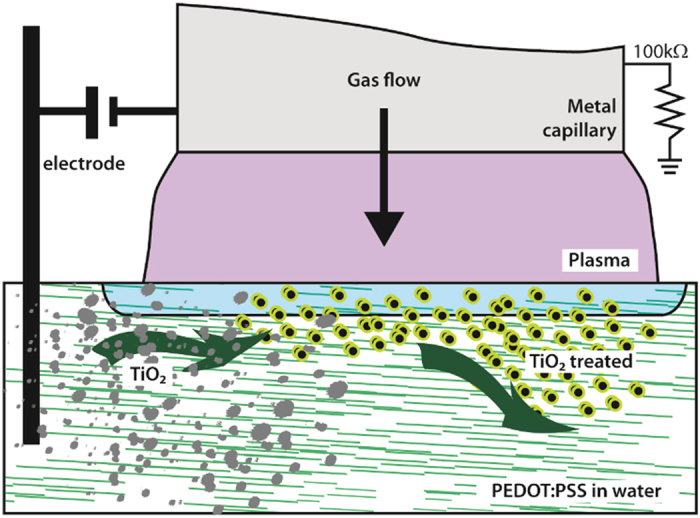
Schematic diagram depicting the plasma set-up.

An atmospheric pressure direct-current (DC) plasma was ignited between a stainless steel capillary (with an internal diameter of 0.25 mm and an external diameter of 0.5 mm) and the surface of the TiO_2_/polymer colloid, Fig. 1. The inert gas flow (pure He) was held constant at 25 sccm. The experimental set up has also been described in detail elsewhere^23^,^24^. The distance between the capillary and the plasma–liquid interface was initially adjusted to 0.9 mm. A carbon rod was used as a counter electrode (anode) and kept at a distance of 2 cm from the metal capillary (cathode), which was connected to earth via a 100 kΩ resistor. Microplasma processing was carried out, in open air, for three consecutive periods of 10 minutes. The initial voltage was 1.63 kV and this was progressively reduced to 0.54 kV to maintain a constant current of 0.5 mA. The pH of the solution changed slightly from the starting level of 4.5 to 4.7 after processing.

Fourier transform infrared spectroscopy (FTIR, Perkin-Elmer Spectrum 2000) was used to determine the surface terminations of the TiO_2_. UV-Vis spectroscopy (Perkin-Elmer Lambda 35) was used to characterize the optical properties of the samples. Transmission electron microscopy (TEM) was performed using a JEOL JEM-2010. The NPs were also investigated before and after processing using scanning electron microscopy (SEM, FEI Quanta 2003D). In order to study the electrical properties of the TiO_2_/PEDOT:PSS nanocomposites, 40 μL samples were drop-cast onto inter-digitated gold electrodes on glass and allowed to dry for 6 hours at room temperature before measurement. The test structures consisted of 20 pairs of electrodes with a 200 μm spacing, covering an area of 6 mm diameter. The film thickness was ~150 nm. DC current-voltage (IV) plots were obtained using a SemiLab DLS-83D DLTS semiconductor test system with an electrometer capability in the nA range. IV measurements represent the average value across the films.

## Results

**Figure 2:**
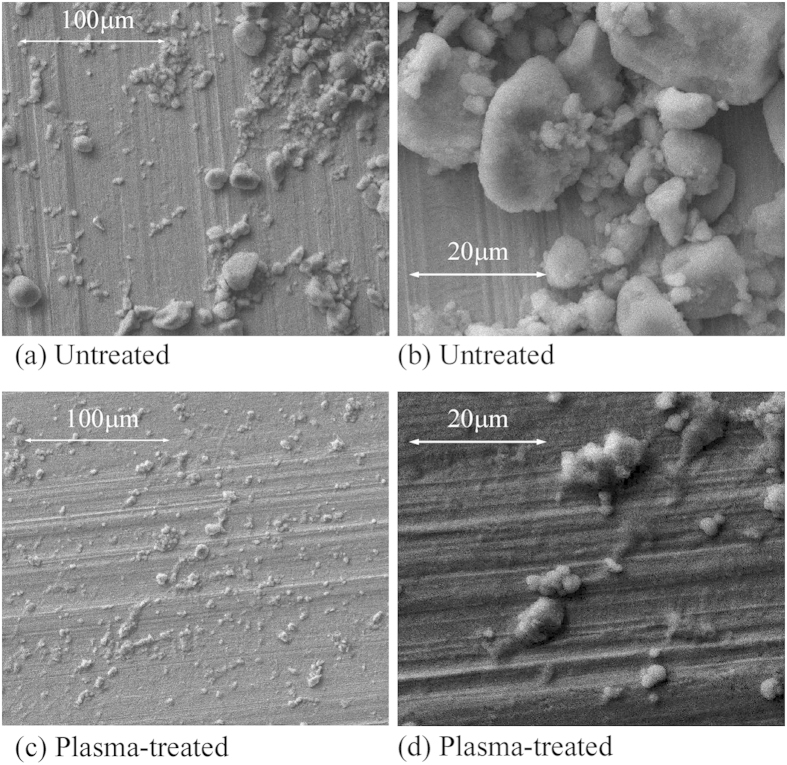
SEM of TiO_2_ NPs in PEDOT:PSS (**a,b**) untreated, and (**c,d**) plasma-treated.

**Figure 3:**
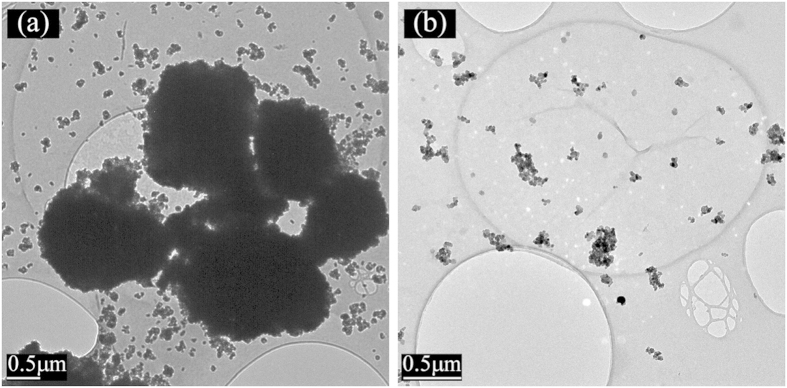
Typical TEM images of (**a**) untreated and (**b**) plasma-treated TiO_2_ /PEDOT:PSS samples.

For the plasma treated sample, TiO_2_ NPs remain stably dispersed throughout the time (a few hours) required for characterisation and analysis, as detailed below, while over the same period, sedimentation of particles is clearly visible for the untreated samples. Some particle sedimentation has been observed, after two days, for the plasma-treated samples. The SEM images of the untreated and plasma-treated samples are shown in Fig. 2a–d. For the untreated sample (Fig. 2a,b), large agglomerates (10–20 μm) with irregular shapes are common, whereas with the plasma-treated samples, Fig. 2c,d, all 10–20 μm agglomerates, observed in the untreated case, have been broken down into much smaller agglomerates (~1 μm and smaller) which appear to be uniformly dispersed within the sample. These particulates consist of multiple 25 nm diameter primary NPs. The improved dispersion and reduced agglomeration is clearly visible in SEM and TEM images, Fig. 3.

TEM analysis has been carried out to further investigate the particle/polymer interaction. Untreated and plasma-treated colloid samples were drop-cast on to TEM sample grids and dried overnight. Figure 3 a shows a typical image of an untreated sample where thick patches (>10 μm) are seen as a result of heavy agglomeration, whereas such features have not been found in the plasma-treated samples. Instead, particles appear to be a lot better dispersed.

**Figure 4:**
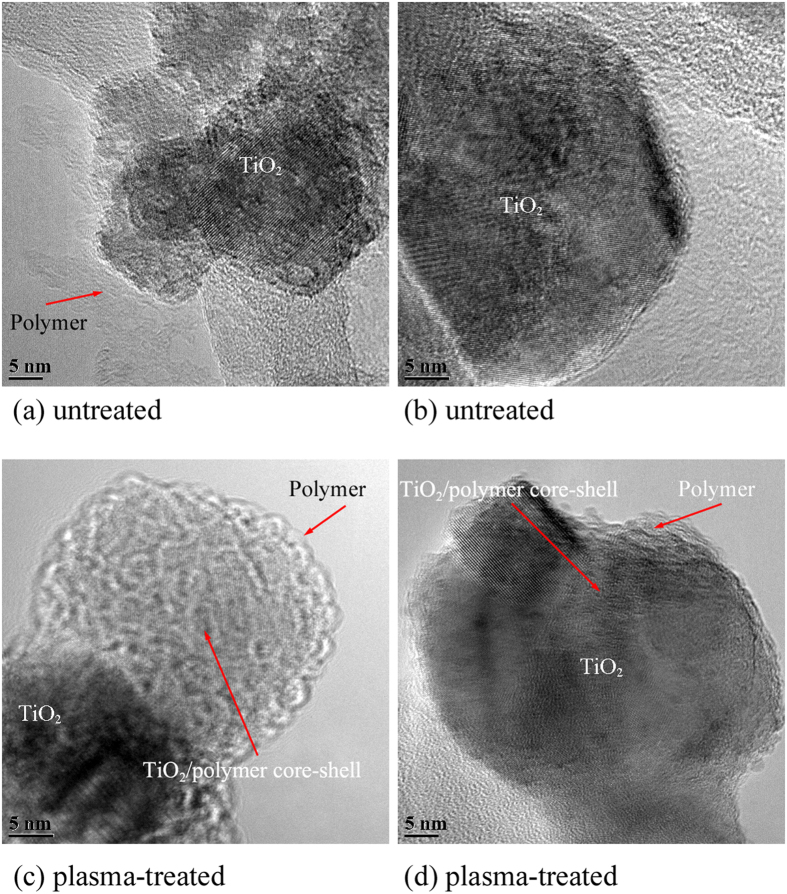
Typical high resolution TEM images showing details of polymer/TiO_2_ interface: (**a,b**) untreated sample and (**c,d**) plasma-treated sample.

In Fig. 4 the high resolution TEM images provide details of the polymer/TiO_2_ NP interface. For the untreated sample, the analysis over a range of NPs has consistently shown an amorphous polymer region which appears to be segregated from the NPs rather than uniformly encapsulating them (Fig. 4a). Also in some cases, the polymer coating in untreated samples is much less visible (Fig. 4b), indicating weak polymer adsorption on the TiO_2_ surface. On the other hand, for the plasma-treated sample (Fig. 4c,d), each of the NPs under observation displayed a TiO_2_ core/polymer shell structure; that is, the TiO_2_ NPs have been encapsulated by a layer of polymer. In particular, Fig. 4c displays crystalline fringes of a TiO_2_ NP (lower left) surrounded by an amorphous layer, which is believed to correspond to the polymer encapsulation. On the top right of Fig. 4c the focus of the TEM image is on the surface of a TiO_2_ NP so that it exhibits the amorphous structure of the polymeric shell. In Fig. 4d, this is also clear as the crystalline structure as well as the amorphous shell are both in focus.

**Figure 5:**
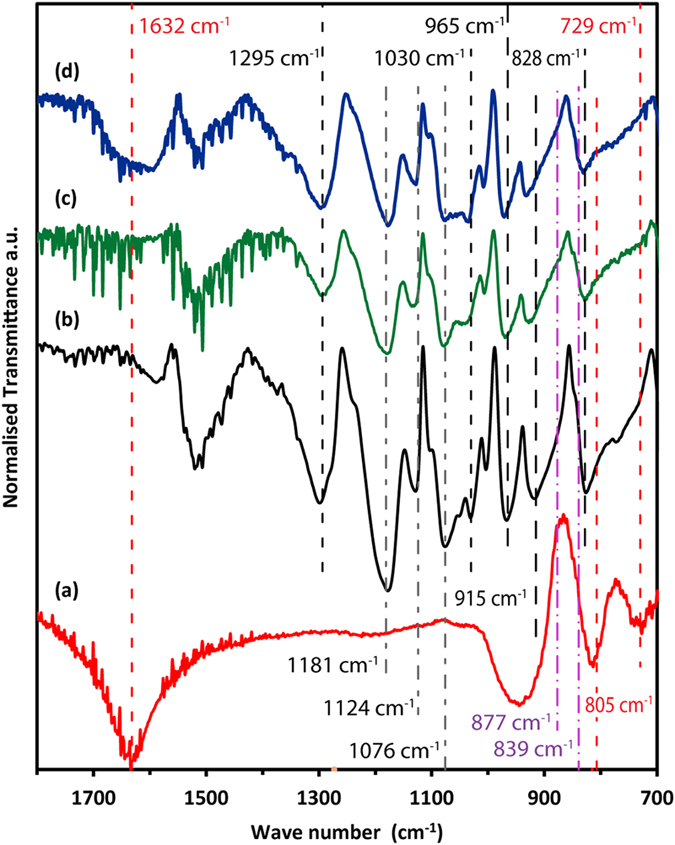
Normalized FTIR spectra with baseline correction (**a**) TiO_2_, (**b**) PEDOT:PSS, (**c**) untreated TiO_2_/PEDOT:PSS, (**d**) plasma-treated TiO_2_/PEDOT:PSS. The characteristic peak locations expected for TiO_2_, PEDOT:PSS and H_2_O_2_ are labeled in red, black and purple dashed lines, respectively (colour plot available online).

FTIR results of both plasma-treated and untreated samples are shown in Fig. 5. The FTIR signals of pure TiO_2_ (dry powder, red curve) and PEDOT:PSS (black curve) are also shown for comparison. For all the spectra, the transmission has been normalized to the strongest absorption and baseline correction was also applied. All the samples were drop-cast on silicon substrate and dried for 4 hours before analysis. It can been seen that the FTIR spectrum of pure TiO_2_ shows the typical characteristic peak at 805 cm^−1^ for Ti-O^25^ and 729 cm^−1^ for O-Ti-O bond^26^ vibration. The peak at 1632 cm^−1^ is caused by the O-H bending vibration due to water trapped between NPs possibly in the form of surface coordinated water^27^.

The polymer FTIR signal (black in Fig. 5) shows its typical signatures. For instance, the peaks at 965 cm^−1^, 915 and 828 cm^−1^ can be assigned to the C-S bond of the thiophene ring in PEDOT^28^, the S = O vibration near 1295 cm^−1^, and the O-S-O signal at 1030 cm^−1^ are identical to those for the sulfonic acid group of the PSS chain. The peaks at 1181 cm^−1^, 1124 cm^−1^ and 1076 cm^−1^ can be attributed to C-O-C bond vibration^29^. For the untreated sample (green curve in Fig. 5), in which TiO_2_ particles are dispersed in the PEDOT:PSS/water solution, some characteristic peaks of PEDOT:PSS can been seen. The peak at 729 cm^−1^ of bulk TiO_2_ is small but still visible, while the 805 cm^−1^ absorption might be masked by the polymer signal. The plasma-treated NPs (blue in Fig. 5), as observed with untreated NPs, still show the peaks at 729 cm^−1^ and the 805 cm^−1^ of bulk TiO_2_; the first is weak and the second overlaps with polymer absorption. The sample does not show any absorption above 3000 cm^−1^, however the peak at 1632 cm^−1^ has reappeared (overlapped with polymer absorption).

**Figure 6:**
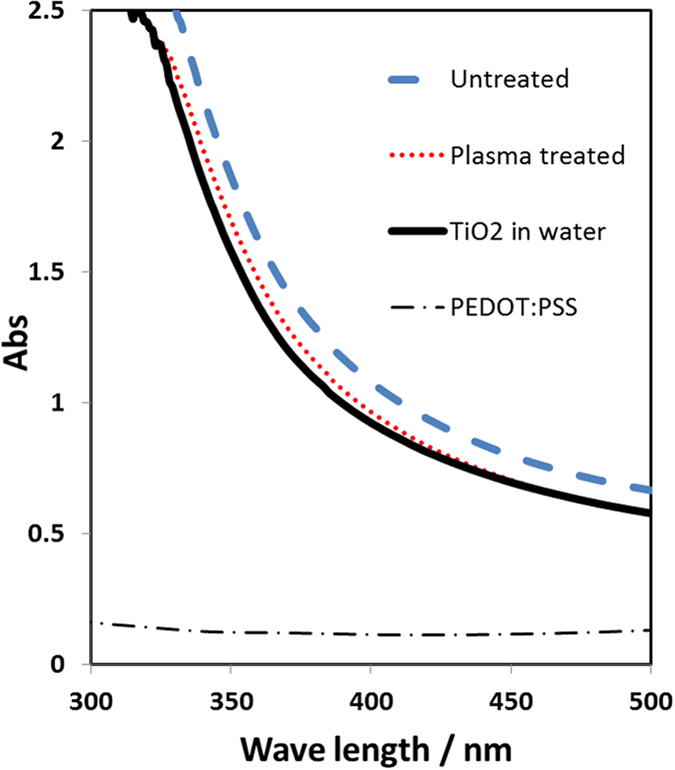
UV-vis absorption spectra of TiO_2_/PEDOT:PSS samples.

Figure 6 shows the TiO_2_/PEDOT:PSS colloidal absorption spectra before and after plasma treatment. Using pure water as background for all samples, pure PEDOT:PSS has a limited contribution to UV absorption below 400 nm, and has no absorbance for the visible range (>400 nm). The addition of TiO_2_ increases the absorption in the UV range (up to 400 nm) due to the strong UV absorbing properties of the NPs. The absorption of TiO_2_/PEDOT:PSS samples (both plasma treated and untreated) exhibited minor change compared to unmodified TiO_2._

**Figure 7:**
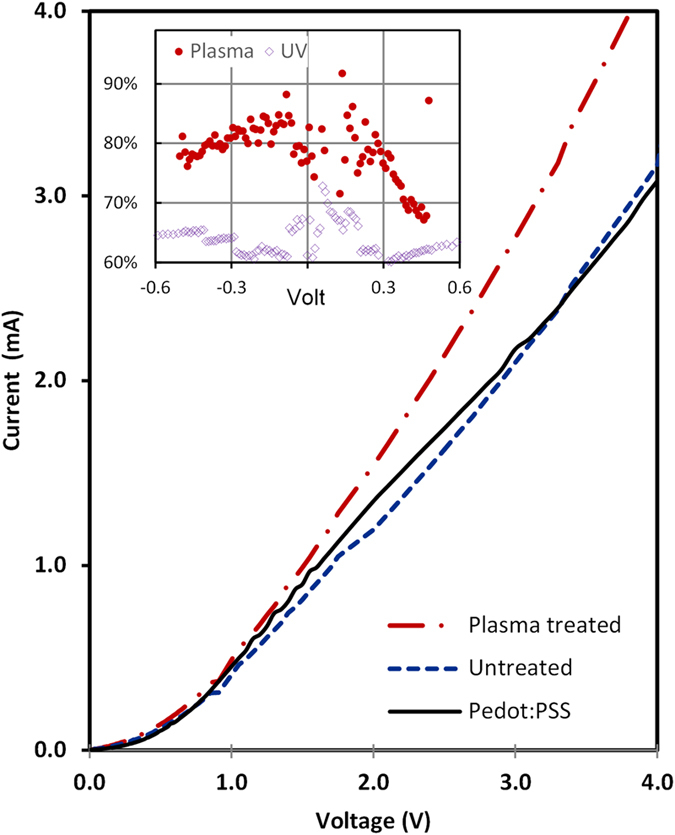
Current−voltage (I−V) characteristics of PEDOT:PSS polymer (dotted), untreated (blue) and plasma treated TiO_2_/polymer nanocomposites (red).

The dark current−voltage (I−V) characteristics of pure PEDOT:PSS (nominal conductivity specification of 1 Scm^−1^), untreated and plasma-treated TiO_2_/polymer nanocomposites are shown in Fig. 7. Each sample has been drop-cast onto inter-digitated gold electrodes on glass and allowed to dry for 6 hours before measurements. The conductivity is measured predominantly in the horizontal direction with coplanar electrodes and the composite I-V characteristics are symmetrical and near-linear except around 0 V. The plasma treatment of pure PEDOT:PSS without nanoparticles results in a decrease in conductivity by up to 20%, Fig. 7 (inset), compared to ~35% reduction after UV exposure. The addition of TiO_2_ NPs to polymer impacts film conductivity to varying degrees. The untreated sample exhibits similar or slightly lower conductivity compared to native polymer whereas after plasma treatment, a significant increase in film conductivity, up to 40%, is observed.

## Discussion

The enhanced dispersion of TiO_2_ NPs in polymer with negligible re-agglomeration has been achieved using direct plasma exposure of the colloid solution, as evidenced from the SEM and TEM images, prior to film formation. This contrasts considerably with the agglomeration observed in untreated samples and with examples reported in the literature^30^. The enhanced conductivity observed with the inclusion of a significant concentration of plasma-treated TiO_2_ NPs provides strong evidence to support the TEM observations and the conclusion that an intimate inorganic-polymer binding has resulted from plasma-induced reactions in the colloid. TiO_2_ has a natural tendency to agglomerate especially for smaller particles^8^, to the extent that sonication has limited efficacy^10^. Immersion in a low ionic strength (IS) solution can help ameliorate this situation by increasing the electrostatic repulsion through enhanced zeta potential and double layer thickness. However the hydrodynamic diameter increases rapidly with IS. Electrostatic stabilisation is possible by adjusting pH values well away from the isoelectric point. Both the pH of native PEDOT:PSS and the initial pH of the TiO_2_/PEDOT:PSS solution were 4.5. After plasma treatment the pH of the solution increased slightly to 4.7. Depending on IS, this is within the pH range where electrostatic stabilisation is less effective, and in other reports, pH values around 2 were typically required for NP stabilisation in PEDOT:PSS^31^,^32^,^33^,^34^. The plasma treatment has clearly shown the ability to improve the dispersion of the NPs. We have observed this effect for a range of analogous processes and with various nanomaterials^15^,^35^. The nature and mechanism by which the “fragmentation” of agglomerates is achieved, remain unclear however. Chemical interactions through plasma-produced radical species, as well as electrostatic interactions due to the presence of ions/hydrated electrons, may be responsible. The formation of a TiO_2_/polymer core shell structure observed after plasma treatment is likely to be due to an enhanced electrostatic interaction between polymer and TiO_2_ NPs induced by plasma processing. FTIR analysis has confirmed that the spectra of PEDOT:PSS/TiO_2_ samples contain contributions from both TiO_2_ and PEDOT:PSS. The IR bands for O-S-O (1030 cm^−1^) and C-S-C (965 and 915 cm^−1^) vibration^28^ for untreated sample have been shifted to higher wave numbers (1040 cm^−1^ for S-O-S, 971 cm^−1^ and 925 cm^−1^ for C-S-C) for the plasma treated sample, indicating stronger O-S-O and C-S-C bonds in the polymer with the presence of TiO_2_.

Chemical reactions are initially induced at the plasma-liquid interface where a range of radicals/compounds and hydrated electrons are produced or absorbed from the gas phase. These are generally due to interactions of the plasma with water molecules and although direct plasma-polymer or plasma-NPs interactions are in principle possible, they remain highly unlikely due to the colloid being mainly composed of water. From these primary chemical products a range of cascaded reactions are possible affecting in different ways the dispersed components, i.e. polymer and NPs. Therefore the surface chemistry of the TiO_2_ NPs is expected to derive directly from plasma-water induced reactions with negligible effects due to the presence of the polymer, as long as the polymer concentration is kept relatively low. Plasma-water interactions are currently the subject of specialized experimental and theoretical efforts^36^, and the direct measurement of OH and other plasma-generated radicals involves complex measurements which, to date, have not produced accurate results. Nevertheless the plasma production of OH radicals and H^+^ ions is well established^37^,^38^. Plasma-water interactions have been also found to produce H_2_O_2_^39^, however interaction between TiO_2_ and H_2_O_2_ is not thought to be significant in our case as the characteristic peaks of H_2_O_2_ at 839 cm^−1^ and 877 cm^−1^ 40 were not observed. This finding is consistent with our previous studies where we compared H_2_O_2_ processing alone versus plasma-liquid processes for silicon NPs with^41^ or without a polymer in the aqueous solution^42^.

From the FTIR spectra, the sharp-peak seen at 1630 cm^−1^ for powder TiO_2_ is indicative of weakly bound hydrogens on neutral (i.e. uncharged) surfaces^43^. This is also confirmed by the typical O-H absorption of water that gives rise to a broad peak above 3000 cm^−1^; in particular the surface charges produce a local electric field that affects the orientation of water molecules so that the peak tends to shift to higher wavenumbers (>3500 cm^−1^) for negatively charged surfaces and to lower wavenumber (<3200 cm^−1^) for positively charged surfaces. However the peak at 1632 cm^−1^ is generally much weaker than the 3000–3600 cm^−1^ absorption for trapped water and therefore we expect that the contribution for this absorption also comes from O-H vibration in Ti-OH terminations^27^, which may also explain the broad absorption above 900 cm^−1^. We can therefore conclude that pristine NPs present typical Ti-O-Ti surfaces with in part Ti-OH terminations and also adsorbed trapped water. TiO_2_ surface groups can be either singly, doubly or triply coordinated (TiO, Ti_2_O, Ti_3_O), having a lower coordination with Ti^4+^ than the oxygen atoms in the solid^44^. The missing charge may be compensated by the uptake of one (Ti_2_O(H)) or two protons (TiOH(H)). It has been reported that TiO_2_ NPs dispersed in water are initially covered by hydroxyl group and different surface interactions can take place under acidic or alkaline conditions^45^. With respect to polycrystalline TiO_2_ i.e. P25 (rich in anatase) as used here, the Ti_3_O surface oxygen atoms cannot be protonated in our tested pH range and therefore are not active with respect to the charging behaviour^46^. The FTIR spectrum of pure TiO_2_ shows the typical characteristic peaks at 805 cm^−1^ for Ti-O^25^ and 729 cm^−1^ for O-Ti-O bond^26^ vibration. These peaks are weaker in composite samples due to the presence of the polymer which reduces the FTIR light penetration depth in the film. When TiO_2_ NPs are dispersed in aqueous medium a reduction of the peak at 1632 cm^−1^ is observed indicating the deprotonation of Ti-OH terminations and a reduction in the number of surface trapped water molecules due to the hydrophobic character of resulting TiO^−^ surfaces. However, after plasma treatment the peak at 1632 cm^−1^ reappears (overlapped with polymer absorption) suggesting a re-protonation of some Ti-O sites. This increase in the surface density of Ti-OH terminations are, we believe, due to the presence of OH radicals and H^+^ ions from the plasma where the former cleave Ti-O-Ti bonds and the latter react to form Ti-OH via TiO^−^ + H^+^ → Ti-OH. Due to the high surface density of Ti-OH, condensation of adjacent terminations forms Ti-O-Ti surfaces. Depending on the IS of the PEDOT:PSS/H_2_O, at a pH of 4.7 we would expect significant protonation of TiO sites although most would still remain non-protonated (therefore negatively-charged). Ti_2_O sites are however almost fully protonated (positively-charged). At low pH, the positively charged protonated sites, Ti_2_OH and TiOH, would therefore dominate and could electrostatically enhance the negatively-charged PSS species bonding. Also the hydrophilic character of protonated surfaces would enhance the polymer – nanoparticle interaction.

In this work, low conductivity (~1 S cm^−1^) native treated PEDOT:PSS (no additives) was used to as starting material to highlight the possible impact of NP addition on conductivity which can be expected to be sensitive to the NP dispersion and the quality of the interface with the polymer. The NP fraction was ~75% wt., much higher than typically reported (20% wt.)^17^,^47^. However, the resultant dispersion and smooth surface, as observed in Fig. 2, is a strong indicator that polymer disruption and the formation of significant NP networks has not occurred. The use of TiO_2_^31^ and other n-type NPs (e.g. α-Fe_2_O_3_^32^) for conductivity modulation of PEDOT:PSS has been reported along with the tailoring of TiO_2_ electronic properties (reduced bandgap, fermi-level shifting) through surface modification with polymer/dye interfaces^33^,^48^ and doping^16^. It is unlikely that significant electronic structure modification has occurred in our case due to plasma treatment as very minor change (except intensity) was observed in UV-vis absorption spectra. Yoo *et al.*^30^,^47^ directly mixed TiO_2_ NPs with PEDOT:PSS to form a densely agglomerated composite film displaying diode-like characteristics, with an increase in conductivity for forward bias only, similar to a pure NP film. Sakai *et al.*^31^ reported no conductivity changes for PEDOT-PSS/TiO_2_ NP composite films, fabricated with layer-by-layer assembly, except after irradiation by light. Semaltianos *et al.*^49^ observed a factor of two increase in conductivity using 10% wt. of ZnO NPs (~5 nm). Sun *et al.*^34^ noted a decrease in PEDOT-PSS conductivity with the inclusion of Fe_3_O_4_ NPs up to 20% wt. which was attributed to the insulating nature of the particles. However Raccis *et al.*^32^ observed that, within a very narrow range of concentrations (~1.2%), the conductivity of PEDOT-PSS could be increased up to eight-fold after NP inclusion. Understanding PEDOT:PSS conduction mechanisms is a significant challenge in the search for enhanced or selectable conductivity and resistance to degradation. PEDOT:PSS conduction is not fully understood but is strongly linked to film morphology, which in spin-cast films is thought to consist of quasi-metallic grains rich in PEDOT surrounded by a semi-insulating PSS shell^50^ or as filamentary PEDOT structures^51^. Experimental observations^52^ indicate these grains to be of oblate spheroid shape, approximately 20 nm to 30 nm in diameter and 4 nm to 6 nm in height formed in layers. Conduction is highly anisotopic due to the much narrower grain-grain gap along the in plane diameter direction, compared to the orthogonal. However, quasi-1D variable range hopping characteristics have often been found to fit the observed anisotropic conductivity which van de Ruit *et al.*^53^ concluded being due to the formation of filamentary PEDOT networks operating close to the percolation threshold. High conductivity PEDOT:PSS^54^, up to 3000 S cm^−1^, is thought to be determined by enhanced hopping conduction between grains due to the greater grain agglomeration and crystal size as a result of additives^51^ or the enhancement of filamentary connectedness well above percolation threshold due to processing^53^. As the PSS content is reduced, an apparent percolation threshold is reached, implying a connected network of highly conducting PEDOT:PSS complexes has been established within the poorly conducting PSS matrix. The number of such connected paths would then determine the macroscopic mobility^51^,^53^. The mobility may also be enhanced via conformational changes, e.g. by adding compounds with two or more polar groups, where the PEDOT chains shift from coiled to expanded-coil or linear form. The microplasma treatment of pure PEDOT:PSS has led to a decrease in conductivity by ~20% which is likely due to low dose UV-exposure from the plasma. We have observed polymer conductivity degradation after direct UV exposure over an extended period, as has been reported previously for UV-exposure in air^55^ (see also Fig. 7. inset). The detailed reaction mechanisms between the plasma induced reactive species and the polymer molecules remain unclear. However it is clear that the enhanced conductivity of the composite film is not due to plasma-induced enhancement of the polymer conductivity alone.

As an n-type conductor in a p-type material, the TiO_2_ NPs would be isolated from the conductive networks and not be expected to automatically enhance the polymer conductivity. Charge confinement on the NP surface^56^,^57^ and the resultant surface electric field extending into the polymer between NPs, would impact on polymer mobility through a complex exponential dependence on field strength^32^. Where the fields are moderate or low, the mobility would be enhanced and the degree of enhancement would be dependent on NP-NP spacing, i.e. volume density. The inclusion of NPs up to 40% by volume in this case, may also facilitate greater network connectivity as the polymer volume is reduced. NP-PEDOT interaction may also lead to conformational changes that affect the intrinsic polymer mobility. Raman studies of ZnO in PEDOT:PSS^49^ indicate a change from a coil conformation to a linear form due to the strongly electronegative oxygen atom of the hydroxyl groups (–OH) on the NPs surface which may form hydrogen bonds with the sulphur cation (S^+^) of the thiophene ring of PEDOT thus weakening the electrostatic interaction between PEDOT and PSS. The experimental evidence for conductivity modification by NPs across different materials is, as outlined above, inconsistent. In this work, plasma-treatment of the TiO_2_/polymer colloid has demonstrated conductivity enhancement at high NP concentrations and this suggests considerable scope for optimising factors such as loadings, in order to tailor conductivity, optical and catalytic properties without disrupting the polymer matrix.

## Conclusion

In this report, we have demonstrated a green and rapid approach to disperse TiO_2_ NPs in conductive PEDOT:PSS aqueous solution. It is found that the use of room temperature atmospheric pressure DC plasma can effectively breakdown TiO_2_ agglomerates and introduce surface modification leading to improved dispersion and stability of TiO_2_ NPs. The enhanced electrical conductivity of plasma treated nanocomposites has been attributed to the better particle/polymer interaction, enhanced particle dispersion and better film mechanical stability. This work reveals the potential of low temperature atmospheric pressure plasma processing in addressing the challenging issue of homogeneous dispersion of inorganic particles into a polymer matrix and tailoring of the nano-to-macro structure of hybrid nanocomposites.

## Additional Information

**How to cite this article**: Liu, Y. *et al.* Enhanced Dispersion of TiO_2_ Nanoparticles in a TiO_2_/PEDOT:PSS Hybrid Nanocomposite via Plasma-Liquid Interactions. *Sci. Rep.* **5**, 15765; doi: 10.1038/srep15765 (2015).

## Supplementary Material

Supplementary Information
