# Supplementary material for: Enhanced Dispersion of TiO2 Nanoparticles in a TiO2/PEDOT:PSS Hybrid Nanocomposite via Plasma-Liquid Interactions
Source: Sci Rep. 2015 Oct 26;5:15765. doi: 10.1038/srep15765 (PMC4620561; doi:10.1038/srep15765)

Enhanced Dispersion of TiO_2 Nanoparticles in a TiO_2/PEDOT:PSS Hybrid Nanocomposite via Plasma-Liquid Interactions

Yazi Liu[1,2], Dan Sun[3,*], Sadegh Askari[4], Jenish Patel[5,4], Manuel Macias-Montero[4], Somak Mitra[4], Richao Zhang[6], Wen-Feng Lin[7], Davide Mariotti[4], Paul Maguire[4]

[1] State Key Laboratory of Pollution Control and Resource Reuse, School of the Environment, Nanjing University, Nanjing 210093, PR China
[2] School of Chemistry and Life Science, Nanjing University Jinling College, Nanjing 210089, PR China
[3] School of Mechanical and Aerospace Engineering, Queen's University Belfast, UK BT9 5AH
[4] Nanotechnology and Integrated Bioengineering Centre (NIBEC), University of Ulster, UK, BT37 0QB
[5] Department of Chemical Engineering, Case Western Reserve University, Cleveland, Ohio, 44106-7217, USA
[6] Department of Chemical and Biological Engineering, Zhejiang University, Hangzhou, 310002, PR China
[7] School of Chemistry and Chemical Engineering, Queen's University Belfast, UK BT9 5AG
*d.sun@qub.ac.uk

Supplementary Materials

Fig. S1: SEM image of TiO_2 particles sonicated in ethanol for 30 min

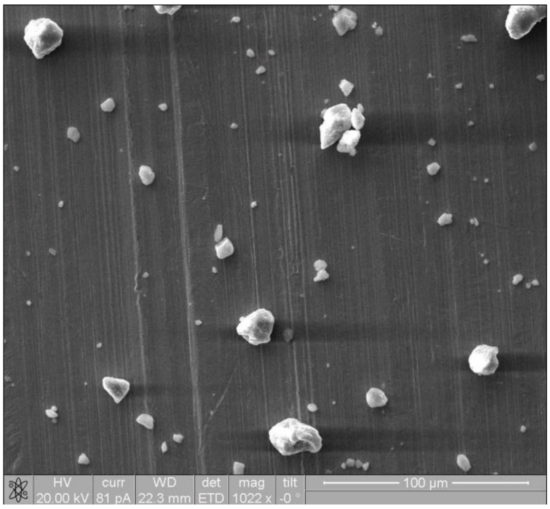

Supplement: Supplementary Information [file srep15765-s1.pdf]
